# DNA building blocks: keeping control of manufacture

**DOI:** 10.3109/10409238.2011.630372

**Published:** 2011-11-03

**Authors:** Anders Hofer, Mikael Crona, Derek T Logan, Britt-Marie Sjöberg

**Affiliations:** 1 Department of Medical Biochemistry and Biophysics, Umeå University, Umeå, Sweden; 2 Department of Molecular Biology and Functional Genomics, Stockholm University, Stockholm, Sweden; 3 Department of Biochemistry & Structural Biology, Center for Molecular Protein Science, Lund University, Lund, Sweden

**Keywords:** Ribonucleotide reductase, RNR, allosteric regulation, specificity site, activity site, ATP cone, dATP inhibition

## Abstract

Ribonucleotide reductase (RNR) is the only source for *de novo* production of the four deoxyribonucleoside triphosphate (dNTP) building blocks needed for DNA synthesis and repair. It is crucial that these dNTP pools are carefully balanced, since mutation rates increase when dNTP levels are either unbalanced or elevated. RNR is the major player in this homeostasis, and with its four different substrates, four different allosteric effectors and two different effector binding sites, it has one of the most sophisticated allosteric regulations known today. In the past few years, the structures of RNRs from several bacteria, yeast and man have been determined in the presence of allosteric effectors and substrates, revealing new information about the mechanisms behind the allosteric regulation. A common theme for all studied RNRs is a flexible loop that mediates modulatory effects from the allosteric specificity site (s-site) to the catalytic site for discrimination between the four substrates. Much less is known about the allosteric activity site (a-site), which functions as an on-off switch for the enzyme's overall activity by binding ATP (activator) or dATP (inhibitor). The two nucleotides induce formation of different enzyme oligomers, and a recent structure of a dATP-inhibited α_6_β_2_ complex from yeast suggested how its subunits interacted non-productively. Interestingly, the oligomers formed and the details of their allosteric regulation differ between eukaryotes and *Escherichia coli* Nevertheless, these differences serve a common purpose in an essential enzyme whose allosteric regulation might date back to the era when the molecular mechanisms behind the central dogma evolved.

## Introduction

**Figure 1:**
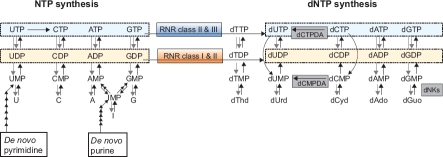
NTP and dNTP synthesis with allosterically/feedback regulated enzymes in dNTP synthesis shown. The class of RNR used, the presence of different dNKs and the choice of dCMP or dCTP for deamination vary between species. Most eukaryotes (e.g. mammals) and gram positive bacteria use dCMP deaminase (dCMPDA) whereas most gram-negative bacteria use dCTP deaminase (dCTPDA). The one-letter abbreviations in NTP salvage synthesis (U, C, A, G and I) stand for both the base and its corresponding ribonucleoside (I stands for hypoxanthine and inosine). Degradation pathways are generally not included with the exception of dephosphorylation events, which are shown by grey arrows unless they directly take part in dNTP synthesis. The dephosphorylations of (d)NTPs to (d)NMPs can both occur as a one-step procedure or via (d)NDPs.

It is critically important that the cell has a tight control over the synthesis of the dCTP, dTTP, dGTP and dATP building blocks (dNTPs) needed for DNA replication and repair since the mutation rate increases dramatically if the levels of the four dNTPs are either unbalanced or generally too high (Mathews, 2006). As illustrated in Figure 1, ribonucleotide reductase (RNR) has a central role in the *de novo* synthesis of dNTPs by making deoxyribonucleotides from the corresponding ribonucleotides (Nordlund and Reichard, 2006). This enzyme is regulated on many levels, including allosteric and transcriptional regulation, cell-cycle specific proteolysis in mammalian cells and by small inhibitory proteins such as Smll and Spdl in yeasts (Nordlund and Reichard, 2006). We will only discuss the allosteric regulation in this review. Three different groups of enzymes in the deoxyribonucleotide synthesis pathway are subject to allosteric or feedback control (Figure 1): RNRs, dCMP/dCTP deaminases (dCMPDA/dCTPDA) and deoxynucleoside kinases (dNKs). RNR is considered to be the master regulator, as verified by numerous studies of mammalian and yeast cell lines where its allosteric sites have been mutated. Many of these cell lines have severely skewed dNTP levels and dramatically increased mutation rates (Weinberg et al., 1981; Chabes et al., 2003; Kumar et al., 2011). Knowledge of RNR's allosteric regulation is also relevant for cancer therapy since many of the currently used anticancer drugs, including gemcitabine, cladribine, fludarabine and clofarabine, mimic the natural substrates and allosteric effectors of this enzyme (Nocentini, 1996; Parker et al., 1991). The synthesis and regulation of the four dNTPs is a sophisticated task and, despite RNR having attained a “scientific age” of over 50 years, research on this enzyme continues to uncover new and fascinating aspects. In particular, two recent studies have contributed intriguing advances in our understanding of the intricacies and complexities of its allosteric regulation (Fairman et al., 2011; Rofougaran et al., 2008).

## Three major RNR classes catalyzing equivalent radical chemistry

The enzymatic reaction catalyzed by RNR involves a transient cysteinyl free radical in the active site, and RNRs have been organized into three main classes and a few subclasses (shown in Table 1) primarily based on how this radical is (re)generated in each catalysis cycle (Nordlund and Reichard, 2006). All classes of RNRs are believed to have evolved from a common ancestor since they share structural features in the active site. One of the most obvious similarities is a 10-stranded α/β-barrel, a unique tertiary structure that RNRs only share with glycyl radical enzymes, which are related functionally to class III RNRs. The mechanism of catalysis and free radical chemistry of the RNR classes and subclasses are well covered by previous reviews (Nordlund and Reichard, 2006; Cotruvo and Stubbe, 2011a) and are therefore only briefly mentioned here. The RNR classes also differ in the nature of the substrate used: class I and some class II RNRs work on 5'-nucleoside diphosphates (NDPs), whereas other Class II and class III enzymes operate at the 5'-nucleoside triphosphates (NTPs) level (Figure 1).

**Table 1:** RNRs are classified into three main classes (I-III) and a few subclasses differing in oxygen dependency, subunit composition, free radical chemistry, and allosteric regulation.

Classes	Genes (subunits)	Free radical chemistry	Allosteric sites	Active form:	Inhibited form
Class I (Ia): *aerobic*	NrdA (R1, α), NrdB (R2, β)	NrdB: Tyrosyl radical and Fe-O-Fe centre.	s-site + a-site (majority)	α_2_β_2_ (general) α_6_β_2-6_ (ATP, eukar.)	α_4_β_4_ (*E. coli*) α_6_β_2_ (eukar.)
Ib	NrdE (R1E, α), NrdF (R2F, β)	NrdF: Tyrosyl radical and Mn-O-Mn (or Fe-O-Fe) centre.	s-site	α_2_β_2_†	None
		NrdI needed for the generation of the Mn-O-Mn centre.			
Ic	NrdA(R1, α), NrdB* (R2, β)	NrdB: Lacks tyrosyl radical. Unpaired e in Fe-O-Mn centre	α subunit associated features are equivalent to canonical class I (Ia) enzymes		
Class II: *oxygen independent*	NrdJ(α)	5'-deoxyadenosyl radical generated from AdoCbl	s-site (rarely a-site)	α (monomeric class II) α_2_ (dimeric class II)	?
Class III: *anaerobic*	NrdD (α)	NrdD: Glycyl radical. NrdG (activase) needed for the generation of the glycyl radical.	s-site + a-site (majority)	α_2_†	α_2_†

* Named as NrdB^phe^, NrdB^Leu^, NrdB^Val^ depending on which amino acid the tyrosyl radical is replaced by.

† Additional complexes formed by the association of RNR subunits with activating components such as NrdI and NrdG are excluded from the table.

Class I RNRs (Nordlund and Reichard, 2006; Cotruvo and Stubbe, 2011a) are predominant in eukaryotes but are also found in several bacteria and viruses, as well as in a few archaea (Lundin et al., 2010). All class I RNRs contain two subunits, generally termed R1 (α) and R2 (β), which are both essential for enzyme activity. The α subunit contains the catalytic site and in most cases two allosteric sites, whereas the β subunit in canonical class I enzymes harbors a tyrosyl radical that is able to generate the active site cysteinyl radical in the α subunit via a long range proton-coupled electron transport chain. Class I is sometimes referred to as the aerobic RNR since it needs oxygen to form a metal centre (an Fe^III^-O-Fe^III^ centre in canonical class I RNRs) which is needed for the generation and stabilization of the tyrosyl radical in the β subunit. Two subgroups of class I enzymes, termed class Ib and Ic (Cotruvo and Stubbe, 201 la), have a metal centre and radical chemistry that both deviate from the canonical class I enzymes (class la). The class Ib RNRs, which are only found in bacteria and bacteriophages, have a Mn^III^-O-Mn^III^/tyrosyl radical centre formed in the presence of the NrdI protein encoded in the class Ib operon. In the absence of NrdI class Ib can also form a canonical Fe-O-Fe/tyrosyl radical centre, but recent biochemical and genetic studies suggest that the Mn-variant is the physiologically relevant form (Martin and Imlay, 2011; Cotruvo and Stubbe, 2011b; Cox et al., 2010; Crona et al., 2011b). In the class Ic enzymes, the tyrosyl radical is replaced by another amino acid (Phe, Leu or Val) and the unpaired electron is provided by their Mn^IV^-O-Fe^III^ metal centre instead. In bacteria, archaea and bacteriophages, the α and β subunits are generally referred to as NrdA and NrdB (class la and Ic) or NrdE and NrdF (Ib).

Class II enzymes are found in bacteria, archaea, viruses, and a few unicellular eukaryotes (Lundin et al., 2010). These enzymes, often referred to as NrdJ, are not dependent on a second subunit for enzyme activity and instead use 5'-deoxyadenosylcobalamin (AdoCbl) for the generation of the cysteinyl radical. The class II RNRs can be subdivided into monomeric and dimeric enzymes with limited sequence homology. The dimeric class II enzymes are more closely related in their amino acid sequence to the class I enzymes than they are to the monomeric enzymes (Lundin et al., 2010).

Class III RNRs, NrdD, are found in bacteria, archaea, bacteriophages and a few eukaryotes (Lundin et al., 2010), They are only active under anaerobic conditions due to their oxygen-sensitive glycyl radical, which most likely comes into direct contact with the active site cysteine during the catalytic cycle (Logan et al., 1999). A second protein (NrdG) is needed for the initial generation of the glycyl radical but once formed, the radical is stable for several catalytic cycles. In eukaryotes the class III gene sequences NrdD and NrdG are fused into a single open reading frame (Lundin et al., 2010).

In addition to the main subunits and free-radical activating proteins (NrdI and NrdG), ribonucleotide reduction also involves factors that reduce the disulfides formed in the α protein during each catalytic cycle. Class I and II enzymes are reduced by thioredoxins and glutaredoxins, whereas the class III enzymes are reduced by formate. Even though thioredoxin has been observed to enhance the interaction of *Escherichia coli* R1 and R2 (Kasrayan et al., 2004), complexes between RNR and glutaredoxin/thioredoxin have not been observed and the redoxins have no obvious effect on allosteric regulation. We will not discuss the reductants further in this review.

Each class of RNR has specific environmental requirements (e.g. oxygen, metal ion, and cofactor availabilities), and many bacteria and archaea encode more than one RNR operon within one and the same species (http://rnrdb.molbio.su.se). Microorganisms that live both aerobically and anaerobically could for example have either a class II enzyme or a combination of class I and III enzymes. An advantage in the latter case is that they do not need AdoCbl for their dNTP synthesis. Interestingly, more than 5% of the fully sequenced bacterial genomes, one archeon, and three unicellular eukaryotes code for all three RNR classes, and it has been suggested that this may be beneficial for growth in rapidly fluctuating environments (Lundin et al., 2010). For P. *aeruginosa*, which encodes all three classes of RNR, it has recently been shown that the three classes are differentially expressed (Torrents et al., 2005; Sjöberg and Torrents, 2011). In eukaryotes, which almost exclusively have the canonical class I RNR, it is common that they have more than one canonical class I variant with different expression profiles. Mammalian cells use one variant of the small subunit (R2) for DNA replication during S-phase, and another variant (p53R2) is expressed constitutively at a low level to supply the mitochondria with dNTPs. The p53R2 protein was originally identified as a DNA-damage inducible protein (Tanaka et al., 2000; Nakano et al., 2000) but later its low constitutive expression was suggested to be important for mitochondrial synthesis (Håkansson et al., 2006). In a seminal study, p53R2 deficiencies were identified in several patients with mitochondrial depletion syndrome (Bourdon et al., 2007).

## General structure and allosteric sites of RNR

The allosteric regulation of RNR as we know it today was first discovered by Reichard and co-workers, who in 1969 laid the basis for the generally accepted mechanisms of both specificity and activity regulation of the *E. coli* class la RNR (Brown and Reichard, 1969b). The specificity site (s-site) determines which substrates are reduced at the catalytic site, whereas the overall activity site (a-site) acts as a master switch that can turn the enzyme on and off. As shown in Table 1, the specificity regulation is widespread across all classes, whereas the a-site predominates in class I (except Ib) and III RNRs. The class Ib enzymes are all truncated in the N-terminus, where the a-site is located, and have accordingly only s-site regulation (Table 1). The class Ic enzymes will be treated as equivalent to canonical RNRs throughout the rest of the text since the Ic classification is entirely based on the β subunit (Högbom et al., 2004).

**Figure 2:**
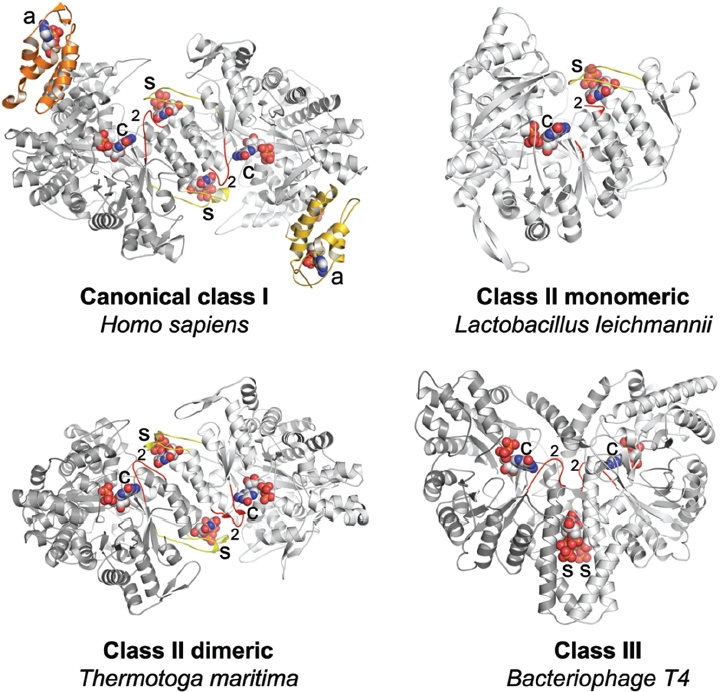
Structures of catalytic subunits from different RNR classes with bound nucleotides highlighted. The a-sites, s-sites, catalytic sites and loop 2 are indicated by the labels a, s, c and 2, respectively. The ATP cones in each α subunit of the human class I enzyme are shown in orange or gold, respectively, whereas it is absent in the representatives from the other classes. Loop 1 and loop 2 are coloured in yellow and red, respectively.

The active form of the catalytic subunit is generally a dimer with the s-sites located in the dimer interface (Figure 2), or is in higher-order complexes composed of several α dimers. The monomeric class II enzymes are an exception to this rule. In this case, the s-site is located within a structural element that mimics the dimer interface found in the other classes (Sintchak et al., 2002). Accordingly, it can have a functional specificity regulation although it is monomeric. In class I enzymes, which in contrast to class II and III enzymes contain both a and β subunits, the holoenzyme is generally described as an α_2_β_2_ complex. Higher oligomers of RNR were first observed as part of the groundbreaking early studies of allosteric regulation of the *E. coli* class la enzyme (Brown and Reichard, 1969a). In analytical ultracentrifugation experiments, a species of about twice the molecular weight of the expected α_2_β_2_ heterotetramer was observed in the presence of dATP. Observations of oligomers were also made on class I RNRs from calf thymus and human tumor cells (Thelander et al., 1980; Cory and Fleischer, 1982), but the concept of higher order oligomerization as a regulatory mechanism languished in obscurity until the 2000s (Kashlan et al., 2002; Rofougaran et al., 2006; Wang et al., 2009; Rofougaran et al., 2008; Fairman et al., 2011). General for all these heavy complexes is that they have only been observed in class I enzymes with a functional a-site. We will return to this theme later in the review.

There are also some special variants of α_2_ and β_2_ structures among canonical class I and II RNRs. In the Aeh1 bacteriophage class I RNR, each α protein is made up of two polypeptides α_a_ and α_b_ (Crona et al., 2011a). In this case, the active enzyme is an (α_a_ + α_b_)_2_β_2_ complex. The class II RNR from *P. aeruginosa* is also split into two polypeptides, NrdJa and NrdJb, and a similar split is also present in the genome of all γ-proteobacteria that encode a class II enzyme (Torrents et al., 2005). *Saccharomyces cer-evisiae* has instead an extra variant of the R2 protein called R4, which is inactive by itself since it lacks important ligating side-chains in its iron center, but is still needed to give the R2 subunit its correct folding (Wang et al., 1997; Chabes et al., 2000). In this case, the active form of the β_2_ subunit is formally a ββ' heterodimer composed of the R2 (β) and R4 (β') proteins.

## Nucleotide pools and general allosteric scheme

The allosteric regulation executed by the s-site prevents each dNTP from increasing too much in concentration in relation to the others. The physiological balance of dNTP concentrations varies from organism to organism: in mammalian cells (Traut, 1994) the dGTP pool is usually by far the lowest (5 μM) followed by dATP (24 μM), dCTP (29 μM) and dTTP (37 μM). In *E. coli*, the dNTP pools are generally 5-10 times higher but in this organism dGTP is also much lower in concentration relative to the other dNTPs (Buckstein et al., 2008). The dNTP concentrations are probably optimized to minimize the mutation rate depending on the affinity of the DNA polymerase for different nucleotides. It has been proposed for the bacteriophage T4 system that the dNTP balance is reflected in the GC content (Hendricks and Mathews, 1997), but this cannot be the explanation in mammalian and *E. coli* cells since the dGTP and dCTP concentrations do not follow each other in these species.

**Figure 3:**
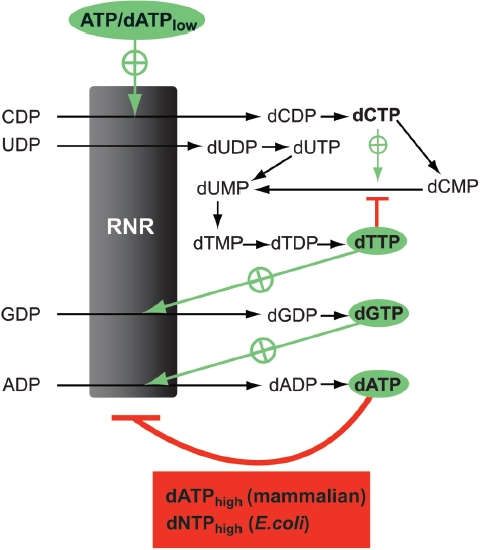
Overview of the allosteric regulation of *de novo* dNTP synthesis. RNR is regulated by four different allosteric effectors (highlighted) and has both a specificity regulation that determines what substrate to reduce (arrows pointed towards RNR) and an overall activity regulation that can turn the enzyme off when there is no need to synthesize more dNTPs (bottom box). Also included in the scheme is dCMP deaminase, which controls the concentration ratio between dCTP and dTTP (the organisms with dCTP deaminase have a similar regulation). Recently, it has been realized that Nature has chosen two different strategies to achieve the overall regulation (bottom box). The *E. coli* enzyme is turned off by high dNTP/ATP ratios (dNTP *=* dATP, dTTP or dGTP), whereas the mammalian enzyme can only be turned off by high dATP/ATP ratios.

The allosteric s-site can bind ATP, dATP, dTTP, and dGTP. As exemplified for mammalian and *E. coli* class la RNRs in Figure 3, ATP and dATP stimulate the reduction of CDP and UDP, whereas dTTP and dGTP stimulate GDP and ADP reduction, respectively (Nordlund and Reichard, 2006). The validity of this regulation has been demonstrated in several ways in cultured cells. One example that illustrates the s-site regulation is when cell growth is temporarily inhibited by hydroxyurea (Skoog and Nordenskjöld, 1971; Hofer et al., 1998), an antiproliferative drug known to inactivate class I RNRs. Directly after the removal of the drug, most dNTPs are very low due to the previous block in their synthesis. A notable exception is dTTP, which remains high since it can be obtained via salvage of thymidine from the culture medium (Bianchi et al., 1986). The first dNTP to increase is dGTP, followed by dATP and finally dCTP. The series of events can be explained by the scheme in Figure 3. At first, when only dTTP is present at high concentration, it will bind to the s-sites and stimulate dGTP production. The dGTP formed will subsequently stimulate dATP production, which finally turns on the synthesis of dCTP/ dTTP. In this way, all four dNTP concentrations are soon in balance with each other and the normal homeostasis is re-established.

The s-site does not bind dCTP efficiently, and additional regulation of dCMP deaminase (dCTP deaminase in *E. coli)* is therefore required to regulate the dCTP/ dTTP ratio (Figure 3). The relative contribution of CDP/ CTP or UDP/UTP as the ultimate source for dTTP synthesis varies between species, with *Trypanosoma brucei* and anaerobically grown bacteriophage T4 as two extreme examples. *T. brucei*, which has very limited CDP/CTP supplies (Hofer et al., 1998; Hofer et al., 2001), lacks dCMP/dCTP deaminase and makes all its dTTP from UDP reduction. The bacteriophage T4 class III enzyme is unable to reduce UTP (Andersson et al., 2000) and uses hydroxymethyl-dCTP instead of dCTP. The dCTP formed in phage-infected cells is efficiently dephosphorylated to dCMP, which is further metabolized to dTTP (Figure 3) and hydroxymethyl-dCTP (Ji et al., 1991).

The a-site can bind either ATP (activator) or dATP (inhibitor) and is needed for turning the enzyme off when the cellular dNTP levels become too high (Nordlund and Reichard, 2006). ATP has similar affinities for both allosteric sites, whereas dATP has a 10-20 times lower affinity for the a-site than for the s-site in the mammalian and *E. coli* enzymes (Brown and Reichard, 1969b; Ormö and Sjöberg, 1990; Reichard et al., 2000). At sub-μM concentrations, dATP acts as a pure s-site regulator and at higher concentrations it binds to both allosteric sites and turns the enzyme activity off. In contrast, ATP has low affinity to both allosteric sites (K_D_ ∼100 μM in *E. coli* class I) but is still able to compete with the dNTPs for binding to the s-site and with dATP for binding to the a-site since it is present at ∼3mM in mammalian and *E. coli* cells (Bochner and Ames, 1982; Buckstein et al., 2008; Traut, 1994). The overall activity regulation of the *E. coli* class la RNR was recently demonstrated to be mechanistically and functionally different from that of the mammalian enzyme (Rofougaran et al., 2008). General inhibition can both occur by dATP alone and by a cross-talk effect between the two allosteric sites where high dNTP levels in combination with an ATP-occupied a-site turn the enzyme off.

During the history of research on RNR allosteric regulation, some variations in the specificity regulation were initially observed in different species, including the *E. coli*, and *T. brucei* class la RNRs, but after more careful scrutiny they can be rationalized in terms of the scheme shown in Figure 3 (Rofougaran et al., 2008; Hofer et al., 1998). One reason for previous misconceptions has been that the substrates have been tested one at a time and often only at a fixed concentration. When analyzing enzyme activity in one-substrate assays, it is easy to come to the wrong conclusion; for example dTTP is able to activate the reduction of all four substrates in *E. coli* when tested one at a time (Brown and Reichard, 1969b) but preferentially reduces GDP in assays when all four substrates are present simultaneously (Rofougaran et al., 2008). A conclusion from these studies is therefore that any deviation reported in the specificity regulation should be verified in this type of assay before being accepted. Another problem with many *in vitro* studies is that they often disregard the fact that under physiological conditions both the a-site (when present) and the s-site are occupied, and the effect of different dNTPs on enzyme specificity should therefore preferably be tested in the presence of ATP. Experiments on the *T. brucei* RNR illustrate the importance of having ATP in the a-site when testing the effect of allosteric effectors on RNR specificity: dGTP was the best inducer of CDP reduction of all effectors when tested alone but the least efficient when tested in combination with ATP (Hofer et al., 1997). The *T. brucei* RNR, was subsequently confirmed both *in vitro* and *in vivo* to follow the specificity regulation scheme shown in Figure 3 but has a deviating a-site regulation and cannot be generally inhibited by dATP (Hofer et al., 1998).

## Specificity regulation - species similarities and differences in specificity and mechanism

The specificity regulation seems to be universal; it exists in all classes of RNRs and only a few members of the Herpesviridae class I enzymes lack it (Averett et al., 1983). It has been shown that herpes viruses have a low-fidelity polymerase, and this is one of the reasons why they are sensitive to many nucleoside analogs as antiviral agents (Hall et al., 1985). Possibly it could be an advantage for herpes viruses to have a high mutation rate in order to evolve more rapidly.

Many RNRs have been crystallized in the presence of allosteric effectors and substrates and from all these studies it has been clear that there is a common theme in the mechanism of specificity regulation. The s-site is in contact with the catalytic site via a flexible loop (loop 2). When the allosteric effector binds to the s-site, it alters the conformation of loop 2 in such a way that it makes the catalytic site more amenable to binding one substrate over the others. The s-site was first localized in the class la enzyme from *E. coli* through the structure determination of the R1 subunit in complex with dTTP and GDP (Eriksson et al., 1997). A set of three loops at the dimer interface (loops 1-3) was seen to respond to dTTP binding by changing conformation relative to the apo state. Loop 2 appeared the most important of the three, as it formed a direct bridge between effector and substrate. Subsequent structural studies of the class Ib RNR from *Salmonella typhimurium* established that the various dNTP effectors induced different conformational changes in loop 2 (Uppsten et al., 2003). However, no substrate complexes were obtained for this system. In addition, the loop movements in the active RNR are most likely dependent on the presence of the R2 protein, and both the *E. coli* and *S. typhimurium* structures were of R1 only.

**Figure 4:**
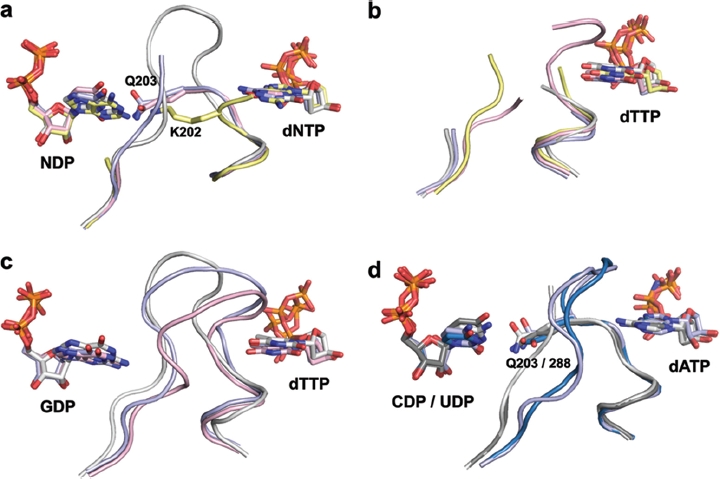
Structural basis for allosteric substrate specificity regulation, (a) The effect of different substrate-effector pairs on loop 2 structure in the *T. maritima* class II enzyme. Nucleotide carbon atoms and loop 2 are coloured grey for the dTTP/GDP complex, yellow for dGTP/ ADP, pink for dATP/CDP and light blue for dATP/UDP. The missing parts of loop 2 in the pink, blue and yellow structures were not visible in the crystal structures and represent unstructured elements. Note in particular the fully ordered loop 2 in dTTP/GDP and the projection towards the substrate of two different hydrogen-bonding residues, K202 and Q203, in dTTP/GDP and dATP/CDP(UDP), respectively, (b-c) Generality across species and RNR classes of the cooperative effect in the binding of dTTP and GDP. The complexes from *T. maritima* class II RNR are coloured grey, *S. cerevisiae* class la in pink, human in light blue and *S. typhimurium* class Ib in yellow (note that there is no dTTP/GDP complex available for *S. typhimurium).* In the absence of substrate, dTTP is unable to structure loop 2 (b) but in its presence (c), loop 2 becomes ordered and forms a cradle around the guanine base of GDP. Only main chain atoms and water molecules are involved in substrate base recognition, (d) Common features of specificity regulation also extend to the dATP/CDP and dATP/UDP complexes shown in light and dark grey, respectively, for *T. maritima*, and light and dark blue respectively for *S. cerevisiae.* Note that a glutamine residue (Q203 in *T. maritima*, Q288 in *S. cerevisiae)* from the effector-proximal side of loop 2 is always projected towards the substrate.

A major breakthrough in understanding of specificity regulation was obtained through a study of seven complexes of the dimeric class II RNR from *Thermotoga maritima* (Larsson et al., 2004). Of these, four were effector/ substrate combinations (dTTP/GDP, dATP/CDP, dATP/ UDP and dGTP/ADP, and three were effector-only (dTTP, dATP and dGTP). This study provided a comprehensive picture of the detailed effects of dNTP-induced conformational changes in loop 2 on substrate binding. Loop 2 can be divided into an effector-proximal side and a substrate-proximal side (Figure 4a). Effector binding induces different conformations of the effector-proximal side of loop 2 that project a variety of main-chain or side-chain atoms from loop 2 towards the substrate, always in a way that matches at least some of the hydrogen-bonding pattern on the substrate base. This work also provided a demonstration that substrate and effector binding were to varying extents cooperative, in line with biochemical results (von Döbeln and Reichard, 1976; Eriksson, 1983; Chimploy and Mathews, 2001; Kasrayan et al., 2004; Crona et al., 2010). dATP and dGTP by themselves structure loop 2 very similarly to the conformation in the substrate complex. However, in the presence of dTTP alone loop 2 was largely unstructured (Figure 4b, grey structure). In this case, the addition of GDP was required to produce a fully ordered conformation (Figure 4a, grey structure). Interestingly, GDP is the only substrate whose base makes interactions with other elements of the active site than loop 2 in the *T. maritima* enzyme, thus it was proposed that GDP could be capable of binding in the presence of a loop 2 conformation that was not preformed (Larsson et al, 2004).

Given the high similarity in the loop 2 sequence between class I and II RNRs from a wide variety of species, it was predicted that the conclusions from the *T. maritima* studies would be general for both classes. Two later studies on class I enzymes confirmed this prediction. A similar series of complexes was determined for the class I RNR from *S. cerevisiae* (Xu et al., 2006) and, very recently, of the human RNR (Fairman et al., 2011). The existence of a large number of structures of class la, Ib and II enzymes from both prokaryotes and eukaryotes enables us now to assess the broader picture and look for similarities and differences between species:

- Loop 2 clearly has a central role in all species, with conformational changes in loop 1 playing a complementary role. With few exceptions, effector base recognition uses main chain atoms only. The cooperative effect of GDP binding on the conformation of loop 2 is also conserved across all species, with dTTP unable to structure the loop in isolation (Figure 4b). When dTTP and GDP are both present, loop 2 is structured, and only main-chain atoms and water molecules participate in recognition of the substrate base (Figure 4c). Other similarities include the fact that the first residue of the effector-proximal side of loop 2 is projected towards the substrate in all dATP/CDP and dATP/UDP complexes (Figure 4d). While the exact details differ, it is evident that the fundamentals of the allosteric specificity regulation have been conserved across class I and II enzymes from evolutionarily very diverse species.
- It should be borne in mind that the final conformations of loop 2 may to differing extents be dependent on the presence of the activating protein (R2 in class I) or cofactor (AdoCbl in class II). When the structure of the class II RNR was determined in the presence of dTTP, GDP and AdoCbl (Larsson et al., 2010), the conformation of loop 2 was seen to be subtly different to that in the dTTP/GDP complex.
- The essentiality of specificity regulation for RNRs is illustrated by the existence of the unusual group of monomeric class II enzymes in which the normally dimerization-dependent regulation is maintained by the insertion of a large structural block within the RNR β-barrel. The crystal structure of the *Lactobacillus leichmannii* RNR showed that the inserted piece contains two α-helices that, together with two other helices, mimic the dimer interface required for specificity regulation, including loop 2 (Sintchak et al., 2002).

Despite significant structural divergence, the class III RNRs provide an interesting study of the conservation of allosteric specificity regulation across large evolutionary distances. To date, only one class III structure is known, that of the enzyme from bacteriophage T4, which has less than 20% sequence identity to the *E. coli* class IRNR. In the T4 enzyme the dimer axis is rotated by 90° relative to that in class I and dimeric class II, with the result that the effector binds in a pocket extending along the length of the helices at the dimer interface rather than being cradled by loops at the ends of the helix bundle (Logan et al., 1999; Larsson et al., 2001). This places the effector base 25 Å from that of the substrate, more than twice as far away as in class I and II. Effector identity is read out entirely by side chains rather than main chain atoms (Larsson et al., 2001). Nevertheless, a series of complexes with four different effectors clearly demonstrated conformational changes in loop 2 that propagate to the part of loop 2 defined as “substrate proximal” in class I and II (Larsson et al., 2001). Thus the allosteric signal, despite being read out and transmitted in a different way, terminates in the same place in all classes (Logan, 2008). The class III RNR is potentially the most promising for structural studies of specificity regulation, since once it has been activated by its activase enzyme it can carry out several reaction cycles in the absence of a cofactor. Thus the conformation of the allosteric loops is most likely not affected by the activase. An additional approach that might contribute to a deeper understanding of the specificity regulation is to compare the allosteric regulation of different RNR classes from the same organism.

## The ATP cone

With the revived interest in the formation of ATP/dATP-induced RNR oligomers, the molecular and structural mechanisms of the overall activity regulation have just started to be revealed (Kashlan et al., 2002; Kashlan and Cooperman, 2003; Rofougaran et al., 2006; Rofougaran et al., 2008; Wang et al., 2009; Fairman et al., 2011; Aye and Stubbe, 2011). The a-site is an ATP cone domain in the N-terminus of the catalytic subunit (Aravind et al., 2000). The signature sequence of the ATP cone (VXKRDG) is present in nearly all canonical class I (including Ic) and III RNRs. In contrast, the ATP cone is very scarce in the class II enzymes and is not present at all in class Ib RNRs (Nordlund and Reichard, 2006). However, an ATP cone is not a guarantee for overall activity regulation since *T. brucei* and bacteriophage T4 class I enzymes, which both contain ATP cones capable of binding dATP, can nevertheless not be inhibited by dATP. Only a few examples of class II enzymes with ATP cones are known, and it is only the one from *Pyrococcus furiosus* that has been verified to have an overall activity regulation (Riera et al., 1997). There are also examples of RNR genes containing several ATP cones in tandem including the class I enzymes from *Chlamydia trachomatis* and *P. aeruginosa* (Roshick et al., 2000; Torrents et al., 2006). Removal of the N-terminal ATP cone in the *P. aeruginosa* enzyme showed that this domain is required for a functional overall activity regulation, whereas the truncated enzyme still could support a functional specificity regulation (Torrents et al., 2006).

**Figure 5:**
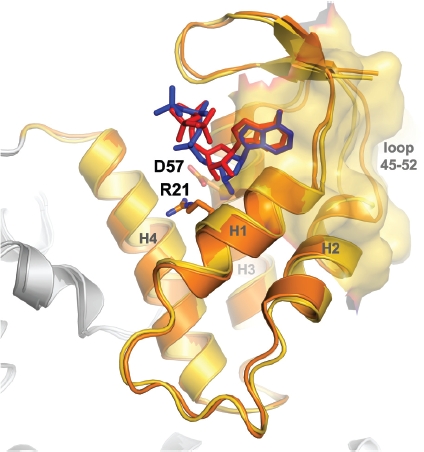
Structure of the ATP cone domain of human class I RNR in complex with dATP (blue) and ATP (red). Residue Asp57, which when mutated eliminates ability to discriminate between ATP and dATP, is shown in stick representation, as is one of its salt bridge partners, Arg21. The helices are labeled H1-H4. The residues involved in the interactions between three α dimers in the dATP-inhibited oligomer (α_6_β_2_ complex) as determined by electron microscopy are shown as a gold surface. The largest differences between the two complexes are in the loop 45-52, which is an important component of the dimer-dimer interface.

The location and structure of the ATP cone domain and the basic binding mode of ATP to it have been known for several years (Eriksson et al., 1997), but it was only very recently that the first crystal structures of dATP and ATP bound to the ATP cone domain of the same RNR were published (Fairman et al., 2011), revealing the structural basis for discrimination between these two nucleotides. The ATP cone domain consists of four helices capped by a short β-hairpin (Figure 5). dATP was seen to bind more deeply in the pocket due to the absence of a 2′-OH group. Residue Ile18 (Ile22 in *E. coli)* was proposed to be essential for this discrimination by acting as a stereochemical barrier to the deeper binding of ATP. When residue Asp57 is mutated to Asn (D57N), this eliminates the ability of the enzyme from mouse to discriminate between dATP and ATP (Weinberg et al., 1981; Eriksson et al., 1981; Caras and Martin, 1988; Reichard et al., 2000). Asp57 makes a salt bridge between two Arg residues in the a-site and possibly hydrogen-bonds to the 2^/^-OH group in the ATP complex. Mutation to Asn would presumably disrupt the electrostatic balance in the binding pocket, resulting in conformational changes that eliminate the ability to discriminate dATP from ATP. Interestingly the largest conformational differences between the dATP and ATP complexes are found in the loop following the second helix of the domain (residues 45-52 in the human enzyme, Figure 5), which is an important part of the interface connecting α-dimers in the dATP-inhibited α_6_β_2_ oligomer (Fairman et al., 2011). While it is not possible at present to correlate these differences directly to the two nucleotide binding modes, it seems very likely that modulation of the conformation of this area is key to the formation of different complexes in the presence of dATP and ATP (see below).

Significant differences are observed in the nucleotide binding modes when comparing the human enzyme with ATP to the *E. coli* R1 structure with bound AMPPNP (Eriksson et al., 1997). ATP binds further out of the pocket in the *E. coli* structure. This has been attributed to a different conformation of the β-cap and an outwards displacement relative to the rest of the ATP cone. The N-terminal portion of the first a-helix in the *E. coli* structure also protrudes further toward the ribose. Since the β-cap is an essential part of the dimer-dimer interface in eukaryotic α_6_ hexamers, one can speculate that subtle sequence-dependent conformational differences in the response to ATP and dATP binding in just this region are responsible for the different oligomerization behaviors of eukaryotic and prokaryotic class I RNRs (see below).

## Multiple mechanisms of overall activity regulation

**Figure 6:**
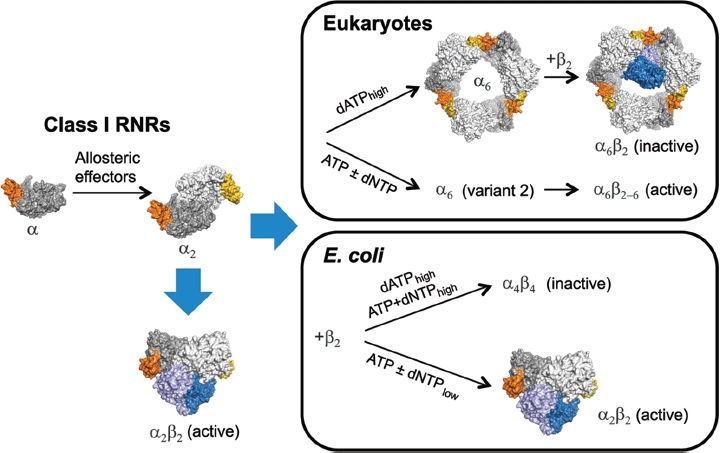
Overall activity regulation in class I RNRs. General for this class is that all allosteric effectors are able to stimulate the formation of α dimers and active α_2_β_2_ complexes. In species with overall activity regulation, heavier complexes are formed in a species-dependent manner. In eukaryotes (mammals and *S. cerevisiae)*, the α subunit can form a hexamer that interacts with the β_2_ subunit to form an inactive α_6_β_2_ or fully active α_6_β_2-6_ complex (higher activity than α_2_β_2_) depending on whether dATP or ATP is bound to the a-site. In *E. coli*, the α_2_β_2_ complexes can bind to each other in the presence of dATP or effector combinations of ATP and high concentrations of dNTPs and thereby form an inactive α_4_β_4_ complex. The protein structures shown are α/α_2_ (human), α_6_ (*S. cerevisiae*), α_6_β_2_ α_6_(*S. cerevisiae*, based on cryo-EM structure) and a model of the α_6_β_2_ complex from *E. coli* built from separate structures of α_2_and β_2_(Uhlin and Eklund, 1994).

Overall activity regulation of class I enzymes seems to be generally dependent on the formation of α_n_ and α_n_β_n_ oligomers. Allosteric effectors that only bind to the s-site stimulate the α subunit to dimerize and interact with the β_2_ subunit to form an active α_2_β_2_ complex (Figure 6). In the physiologically relevant situation where both allosteric sites are occupied, and in the presence of physiological concentrations of α and β subunits, the mammalian RNRs form an α_6_β_2_ complex that is either active or inactive depending on whether ATP or dATP is bound to the a-site (Rofougaran et al., 2006). Until recently, it has been an enigma how ATP and dATP can induce the same quaternary structures but have opposing effects on enzyme activity. A low-resolution x-ray structure of the dATP-bound hexamer from *S. cerevisiae* begins to shed light on this problem by showing that it has a ring-like structure built up from three α dimers (Fairman et al., 2011). When a point mutation (D16R) in the observed interaction interface between each dimer was introduced, the resulting protein was unable to form hexamers in the presence of dATP but could still form hexamers in the presence of ATP. This speaks strongly in favor of the dATP- and ATP-induced complexes being structurally different. A cryo-electron microscopy (cryo-EM) study of the inactive α_6_β_2_ complex (formally α_6_ββ’ complex) showed that the β_2_ subunit was bound in the centre of the dATP-induced hexamer ring (Fairman et al., 2011) and it was suggested that it is locked in a position in which it cannot interact properly with the α subunit to form a functional electron transport chain. The positioning of β_2_ on the inside of the ring also excludes stoichiometries for the inactive complex other than α_6_β_2_, since only one β dimer can be accommodated inside the ring.

In contrast, very little is known about the ATP-induced α hexamer. Preliminary EM data show that a ring is also formed in the presence of ATP (Johansson & Logan, unpublished results) but the structural differences to the dATP-induced hexamer have not yet been studied. However, gel filtration studies of the ATP-induced α hexamer show that it can bind up to three β dimers if the substrate analog gemcitabine-5'diphosphate is added to the enzyme (Wang et al., 2009). Since it would not be possible to fit three β dimers in the centre of a hexamer ring of the type induced by dATP, the ATP-induced hexamer thus appears to have a quite different structure. The α_6_β_6_ form was predicted already in 2001 but was at that time not experimentally verified (Kashlan et al., 2002).

What is then the dominating RNR structure under *in vivo* conditions? Most *in vivo* studies have so far been performed on mammalian and yeast cells with the D57N mutation in the R1 a-site. The strongly increased dNTP pools in the mutated mammalian cell line (dGuo-200-1) as compared to the corresponding wild-type cell lines indicate that quite a large proportion of RNR is normally in its inhibited form (Weinberg et al., 1981). The D57N mutation caused increased dNTP pools also in yeast, but the difference was less pronounced than in mammalian cells under normal growth conditions (Chabes et al., 2003). It has not been possible to study directly what quaternary structure the enzyme has inside the cells, but as mentioned previously, it has been clearly shown that dATP inhibition cannot occur when the surfaces keeping the α hexamer together are mutated (Fairman et al., 2011). It is therefore likely that the inhibited form is also an α_6_β_2_ complex *in vivo.* Similarly, it has not been possible to study directly what the main active RNR form is *in vivo*, but gel filtration and gas-phase-electrophoretic mobility macromolecule analysis (GEMMA) experiments show that at physiological concentrations of the RNR subunits and ATP, the a hexamer strongly predominates over the dimer, and that it mainly forms an α_6_β_2_ complex with the β subunit under these conditions.

Interestingly, the inhibition mechanism of the *E. coli* class la RNR differs from that of the mammalian and yeast enzymes in several ways (Rofougaran et al., 2008). The *E. coli* enzyme has the ability to switch between an active α_2_β_2_ form induced in the presence of ATP, dGTP, dTTP or low concentrations of dATP and an inactive α_4_, β_4_ form induced by high concentrations of dATP or combinations of dGTP/dTTP + ATP (Figure 6). In this RNR, the s- and a-sites communicate with each other to induce the formation of the inhibited complex. Cross-talk effects between the allosteric sites were observed already during the initial studies of the enzyme (Brown and Reichard, 1969b), but the key role of the s-site in this regulation was not realized until recently. This was shown in experiments where ATP was combined with dTTP or dGTP, two allosteric effectors that can only bind to the s-site (Rofougaran et al., 2008). When ATP is used alone, it binds both allosteric sites and stimulates CDP and UDP reduction. When ATP is combined with intermediate dGTP or dTTP concentrations, the enzyme is still active but switched to ADP or GDP reduction, respectively. However, when saturating concentrations of dGTP or dTTP are combined with ATP, the enzyme forms a generally inhibited α_4_β_4_ complex that cannot reduce any of the four substrates. In contrast, the mammalian enzyme has a different allosteric regulation where ATP is always an activator when it binds to the a-site regardless of which other nucleotide is present at the s-site. The difference between the species was demonstrated in experiments where increasing concentrations of ATP were added to a reaction in which dTTP-induced GDP reduction was studied (Rofougaran et al., 2008). The activity of the mammalian RNR was increased 2.5-fold whereas the *E. coli* enzyme was nearly completely inhibited under similar conditions. The inhibition was also strong if other substrates were tested under these conditions.

**Figure 7:**
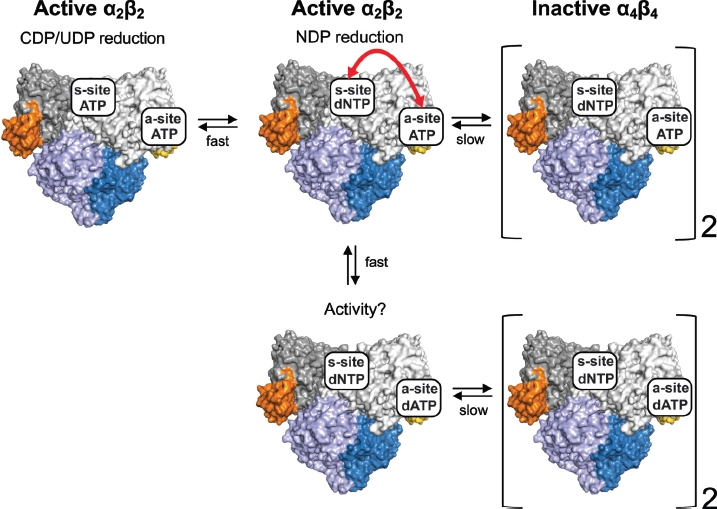
The s-site has a key role in overall activity regulation of the *E. coli* RNR. If the s-site is occupied by a dNTP, a cross-talk signal between the nucleotides in the two allosteric sites (double-sided arrow) leads to the formation of an inhibited α_4_β_4_ complex. At low dNTP concentrations, ATP is able to compete for the s-site and the enzyme rapidly equilibrates between the two top left forms in the figure. At higher dNTP concentrations, the concentration of the form shown in the top middle is high enough to promote formation of the inactive α_4_β_4_ complex (Rofougaran et al., 2008). The lower part of the picture shows that the inhibited form is also formed with dATP in the a-site. However, in this case it is not known whether the intermediate α_2_β_2_ form is active and if a cross-talk signal between the two sites is required for inhibition. Theoretically, a form with dATP in the a-site and ATP in the s-site is also conceivable, but is excluded from the scheme since it is uncertain if ATP ever can compete significantly with binding to the s-site when the dATP concentration is high enough to bind to both sites.

As we have described previously, the ATP concentration is high enough in *E. coli* (∼3 mM) to saturate the a-and s-sites at physiological conditions and, accordingly, a simplified scheme of α_n_β_n_ complexes with fully occupied allosteric sites can be made (Figure 7). It illustrates that the two allosteric sites can communicate with each other in such a way that the ATP-bound a-site is able to sense whether a dNTP or ATP is bound to the s-site. This dNTP can be either dTTP or dGTP and possibly also dATP. However, it is more difficult to verify the effect of dATP in the s-site since it will also compete with ATP for the a-site. The concentration dependence of dNTP + ATP inhibition was interpreted to mean that the formation of the α_4_β_4_ complex is a comparatively slow process in relation to the fast exchange of nucleotides at the allosteric sites. If communication between the sites is disturbed by ATP competing with dNTP for the s-site, the ability of the *E. coli* RNR to form the inhibited α_4_β_4_ complex is perturbed. Note that the equilibrium between the form with ATP in both sites (left panel in Figure 7) and the one with dNTP in the s-site and ATP in the a-site (middle top panel in Figure 7) needs to be very much pushed to the right for inhibition to take place, as evidenced by the experiment described above where increasing concentrations of dTTP/dGTP are added to the ATP-activated enzyme. A nearly complete shift in substrate specificity from CDP/UDP to the particular NDP specified by the dNTP in the s-site therefore occurs before the first signs of inhibition are observed (Rofougaran et al., 2008). A similar inhibition scheme could also in principle be applied to dATP inhibition, but would be less straightforward to verify experimentally since dATP has the highest affinity of all nucleotides to the s-site. Interestingly, the s-site dNTP also had a strong effect on nucleotide affinity for the a-site and in the presence of 2 mM dTTP, ATP inhibited the reaction with a similar IC_50_ value to dATP (Rofougaran et al. 2008). The communication between the two allosteric sites is quite remarkable given the long distance between them.

Another difference between the *E. coli* and mammalian/yeast enzymes is the dependence on the β subunit for the formation of heavy complexes. The mammalian/yeast α subunit can form α_6_ complexes in the presence of ATP or dATP, whereas the *E. coli* α subunit cannot form heavier complexes than α_2_ in the absence of the β subunit. It is unlikely that the *E. coli* α_4_β_4_ octamer will have a structure in which the α subunits build a closed ring like in the eukaryotic α_6_β_2_ complex. In the α_6_β_2_ complex, all the ATP cone domains are in contact with each other, but attempts to model a closed ring from only two *α_2_* dimers lead to occlusion of the β_2_-binding surface (Logan, 2011). Thus the *E. coli* α_4_β_4_ complex must have a different structure. Perhaps this also explains why a tetramers have never been observed in experiments with *E. coli* class Ia RNR (Rofougaran et al., 2008). The contact area between two ATP cone domains is simply too small to stabilize a tetramer in the absence of the β_2_ subunit. However, the relation between oligomerization and allosteric regulation has so far only been studied in a few species and it is not known if the *E. coli* mechanism is widespread in bacteria. A recent comprehensive phylogenetic study suggests that the common ancestor of all eukaryotes inherited its class I RNR genes from bacteria other than alpha- (mitochondrial ancestor) or gamma-proteobacteria (the group to which *E. coli* belongs) but it is not possible to identify exactly from which bacterial family (Lundin et al., 2010). Remarkably, there has also been a late transfer of the class I genes from a eukaryote to a group of the *Bacteriodetes* family (Lundin & al 2010).

In order to better understand when the eukaryote variant of overall activity regulation first appeared in evolution, it would be interesting to study RNRs from a representative of this *Bacteriodetes* group as well as from deeply rooted eukaryotes.

A general question is how many different mechanisms of overall activity regulation there are, and whether there are any universal features common to all enzymes with this regulation. A common theme in the *E. coli* and mammalian canonical class I RNRs is that the interaction between the α and β subunits in the heavy complexes is tighter than in the corresponding α_2_β_2_ complexes (Kasrayan et al., 2004; Crona et al., 2010; Ingemarson and Thelander, 1996; Birgander et al., 2004). A tighter interaction can both be favorable or unfavorable for enzyme activity depending on how the two subunits interact. A tight favorable α-β interaction could possibly explain the high enzyme activity of ATP-induced heavy complexes in eukaryotes.

Much less is known about the mechanisms of overall activity regulation in other RNR classes. The only representative from class III where effector-induced protein oligomerization has been studied is the one from *Lactococcus lactis.* With inhibiting concentrations of dATP, this enzyme seems to form only dimeric complexes both alone and in presence of the activase (Torrents et al., 2000; Torrents et al., 2001). Even less is known about the class II enzymes. Only a few representatives have an ATP cone sequence and the *P.furiosus* RNR is the only one biochemically characterized, which has been shown to be inhibited by dATP (Riera et al., 1997). It is not known whether dATP can induce heavy complexes in this species. In conclusion, several mechanisms of overall activity inhibition exist in Nature, where at least two different oligomerization-dependent mechanisms (canonical class I in eukaryotes and *E. coli* respectively) and one independent (class III in *L. lactis)* have been characterized. Despite the now available eukaryotic class I RNR α_6_β_2_ structure, the mechanistic details of the overall regulation are still intriguing. The regulation similarities within class I RNRs point to shared mechanistic features. However, the different inhibited oligomeric complexes also indicate individual solutions to the same task. In the inhibited α_6_β_2_ complex, the two subunits do not interact to produce a functional electron transport chain. It remains to be shown whether the same is the case for the *E. coli* α_4_β_4_ complex. The class II and III RNRs require neither a second subunit nor a long range electron transport chain for enzyme activity, and can perhaps for that reason not be turned off by oligomerization. As the AdoCbl in class II and the glycyl radical in class III are very close to their active sites, a plausible mechanism could be to change the active site to directly disturb formation of the cysteinyl radical. Given the fundamentally different initiating radical donors in each RNR class and the sparse studies of class II and III overall activity inhibition, there are likely important “cofactor"-adapted regulatory details yet to discover.

## All nucleotide binding sites in RNR communicate with each other

Although the only structurally defined communication between nucleotide binding sites in RNR is the one between the catalytic site and the s-site, there is accumulating evidence that all three nucleotide-binding sites communicate with each other. The long-range communication between the s- and a-sites described above is not unique to *E. coli.* In the mammalian enzyme, it has been shown that mutation of the a-site (D57N) also led to a seven-fold reduction in affinity of dATP for the s-site (Reichard et al., 2000). In other situations, it has been shown that the a-site needs to be occupied with ATP in order to achieve a correct specificity regulation via the s-site (Hofer et al., 1997; Hofer et al., 1998). Further insight into the extensive cross-talk within RNR has been achieved from the studies of how different nucleoside analogs affect the enzyme. Many of the nucleoside analogs used in cancer therapy are able to inhibit RNR by mimicking its substrates or allosteric effectors. Nucleoside analogs such as fludarabine, gemcitabine, cladribine and clofarabine are converted into their corresponding di-and triphosphates by nucleoside/nucleotide kinases and these can subsequently bind to the catalytic and allosteric sites of RNR (Nocentini, 1996; Parker et al., 1991).

An unexpected recent finding was that a hexamers can be induced by clofarabine-5'-diphosphate, a substrate analog that is unable to bind to the allosteric sites (Aye and Stubbe, 2011). One possibility could be that the substrate analog is able to transmit a signal to the a-site that induces the protein to hexamerize.

## Concluding remarks

An ancestral RNR was a prerequisite for the transition from an RNA world to the DNA world more than 3.5 billion years ago. The unique structural core of contemporary RNRs strongly implies that they all have a common origin, but the evolutionary time span has not allowed preservation of distinct amino acid sequence similarities among the contemporary classes of RNR (Lundin et al., 2010). It is therefore interesting to observe that even though RNRs differ substantially in mechanisms to maintain dNTP homeostasis, they still manage to accomplish a common output. Even though comprehensive phylogenetic analyses show that it is not currently possible to identify which of the contemporary RNR classes that is most closely related to the ancestral RNR, we can still make a qualified attempt to order the appearance of the different levels of regulation and substrate phosphorylation levels during the evolution of the RNR family. The ancestral RNR was conceivably a ribonucleoside triphosphate reductase lacking allosteric regulation and working anaerobically, i.e. closer to a class II or III than to a class I RNR. Next to evolve was probably a common feedback control that gradually developed into an allosteric specificity regulation. The overall activity regulation is likely a character that developed later by adoption of the N-terminal ATP cone domain. It is hard to rationalize why some contemporary RNRs among bacteria and archaea seem to manage without this accessory sophistication, but many viruses are known to benefit from less accurate DNA replication mechanisms. It cannot be excluded that the overall activity regulation has evolved more than once, but the strict N-terminal positioning of the ATP cone, when present, speaks for a single event, plausibly with several subsequent losses. What drew the switch from ribonucleoside triphosphate substrates to ribonucleoside diphosphate substrates is not obvious, and many contemporary organisms seem to cope well with triphosphate substrates. The fine-tuned allosteric regulation that RNRs of modern organisms present have developed in several steps, in which allosteric regulation of substrate specificity conceivably preceded development of overall activity regulation, and where ribonucleoside triphosphate substrates conceivably preceded ribonucleoside diphosphate substrates. With billions of years of evolution it is not surprising that several different variants of RNR have evolved and that its allosteric regulation has reached the level of sophistication that we meet in contemporary RNRs.
